# Research on designers’ behavioral intention toward Artificial Intelligence-Aided Design: integrating the Theory of Planned Behavior and the Technology Acceptance Model

**DOI:** 10.3389/fpsyg.2024.1450717

**Published:** 2024-09-16

**Authors:** Jinchuan Jiao, Xiangnan Cao

**Affiliations:** School of Art and Design, Guilin University of Electronic Technology, Guilin, China

**Keywords:** Theory of Planned Behavior (TPB), Technology Acceptance Model (TAM), Artificial Intelligence-Aided Design (AIAD), designer, knowledge level

## Abstract

Artificial Intelligence-Aided Design (AIAD) has numerous advantages and tremendous benefits for designers. However, not all designers are keen to integrate AIAD into their workflow, and their intention to use AIAD remains a research gap. This study explores designers’ adoption of AIAD, utilizing the Theory of Planned Behavior (TPB) and the Technology Acceptance Model (TAM). Drawing on extant literature, we proposed a research model and tested it using data from 392 Chinese designers. The results indicate that in terms of AIAD, (a) designers’ attitudes toward AIAD (*b* = 0.259, *p* < 0.001), subjective norms (*b* = 0.363, *p* < 0.001), and perceived behavioral control (*b* = 0.556, *p* < 0.001) have significant and positive impacts on their intention to use AIAD; (b) perceived usefulness of AIAD (*b* = 0.910, *p* < 0.001) has a positive and significant correlation with attitudes toward AIAD while perceived ease of use (*b* = −0.126, *p* < 0.05) exerts no significant impact on attitudes; (c) the knowledge level of designers (*b* = −0.149, *p* < 0.01) has a negative moderating effect on the impact of attitudes toward AIAD on the intention to use them. The present research then discusses its practical significance.

## 1 Introduction

The advancement of computing power has led to the rapid development of artificial intelligence (AI) technologies, including natural language processing, speech recognition, and machine learning (Sohn and Kwon, 2020). In recent years, various Artificial Intelligence-Aided Design (AIAD) tools have emerged. Examples include Midjourney, an AI-powered art service that enables users to generate images through text inputs (Hanna, 2023), and AIBPS (AI-Based Painting System), a system that can produce numerous images quickly by simulating human drawing processes (Lyu et al., 2022; Xu et al., 2023). These advancements underscore that AIAD can greatly enhance design efficiency and gradually become a part of the design process (Bhattacharya, 2021). However, the adoption of AIAD among designers is not universal, as it is still a novel technology with many limitations. For example, generative AI tools like ChatGPT do not clearly define the boundary between reality and virtual reality (Shin and Ahmad, 2023). The intricate complexity of the algorithms that drive these tools often surpasses designers’ understanding, which could beget designers’ reluctance to use them (Shin, 2024). Therefore, effective functions and intelligible algorithmic logic are essential for a broader application of AI (Shin, 2023). Research regarding the factors affecting designers’ intention to use AIAD can provide valuable insights for the targeted development of these tools. Once designers trust AI services or service providers, they will believe these services are easy to use and continue to adopt them (Shin, 2023). Several prior studies have probed into the determinants of the intention to use AIAD (Du et al., 2023; Xu et al., 2023; Li, 2024; Fan and Jiang, 2024). Du et al. (2023) examined the influences of AI literacy and AI anxiety on attitudes. Xu et al. (2023) explored the positive impacts of hedonic motivations and perceived trust on perceived usefulness and ease of use. Li (2024) discovered that designers’ expectations of AI-generated content’s (AIGC) performance, social influence, and perceived risks are key determinants of their behavioral intentions. Fan and Jiang (2024) revealed the relationships between perceived usefulness, perceived ease of use, perceived playfulness, and designers’ continuance intention to use AI drawing tools. They found that perceived ease of use negatively impacts the intention to use them.

Despite numerous studies on the behavioral intention toward AIAD, research gaps still need to be addressed. First, the results of these studies often differ, which can be further discussed by including boundary factors; second, influences of other essential factors on technology adoption are rarely touched upon, such as consumer knowledge (Schreier and Prügl, 2008). Knowledge refers to an individual’s mastery of specific information on a particular subject, which enables the individual to identify specific opportunities (Venkataraman et al., 1990). In exploring the antecedents of AIAD, a person’s knowledge level represents their familiarity with relevant information in this field (Schreier and Prügl, 2008). Some researchers pointed out that individuals’ behaviors depend on their knowledge of the subject of interest (Swaminathan, 2003; Barrutia and Gilsanz, 2013). To this end, designers’ knowledge level of AIAD could also impact their intention to use AIAD.

The Theory of Planned Behavior (TPB) is a general behavioral theory that explains individuals’ behaviors. It introduces three primary antecedents (i.e., attitudes, subjective norms, and perceived behavioral control) that impact users’ decision-making processes (Ajzen, 1991). Using AIAD can be deemed a general behavior that can be studied using TPB. However, TPB alone cannot thoroughly explain users’ intention to use AIAD, as AIAD consists of novel technologies such as artificial intelligence.

The Technology Acceptance Model (TAM) primarily focuses on how users adopt new information technologies (Davis, 1989). Nevertheless, it does not involve the pronounced features of AIAD, as TAM excludes the fact that it can be used beyond the organization settings and it does not consider other people’s influence on the users’ attitudes and behaviors (Rauniar et al., 2014). Therefore, TAM alone cannot fully explain the intention to use AIAD.

Motivations for users’ intention to use AIAD are influenced by societal norms (subjective norms), perceived behavioral control, and the perceived values (usefulness and ease of use) of novel technologies. Considering the above analysis, integrating TPB and TAM would provide a comprehensive framework for elucidating users’ intentions to adopt AIAD.

After an exhaustive review of extant literature, it is found that TPB has been adopted in AI-related studies (Mohr and Kühl, 2021), and there are studies integrating TAM and TPB to explain the user acceptance and usage intention for services of new technologies (Safeena et al., 2013; Rahman et al., 2017). However, there are few studies integrating TPB and TAM to delve into the antecedents of designers’ intention to adopt AIAD tools (Wang and Chen, 2024). Therefore, by employing both TPB and TAM, as well as individual knowledge, this study probes into the impacts of attitudes, subjective norms, perceived behavioral control, perceived usefulness, perceived ease of use, and knowledge level regarding users’ adoption of AIAD. By combining these two theories and innovatively adding the variable of knowledge level, this study establishes a research framework to explore designers’ adoption of AIAD. The findings presented by this research can serve as references for developing AIAD tools among algorithm detection companies. The present study thoroughly examines the antecedents affecting users’ adoption of AIAD and establishes an integrated structural equation model to evaluate these influences. An empirical study was performed to test the hypotheses, and discussions were held. The organization of this manuscript is as follows: After the first introduction part, the second part is an extensive review of the literature pertaining to AIAD, TPB, TAM, and factors influencing the intention to use AIAD; the third part elucidates the structural equation model and the empirical research conducted; the fourth and fifth parts are dedicated to elucidating the findings and conclusions derived from the empirical research; eventually, the sixth part presents a concluding observation and outlines the potential avenues for future research in AIAD. Our research aims to enhance the understanding of the factors influencing designers’ intention to use AIAD and to further facilitate the adoption of AI-based tools. Specifically, this research aims to answer the following questions: (1) Do the TAM and TPB have good explanatory power in predicting designers’ behavioral intention toward AIAD? (2) How does knowledge level influence designers’ behavioral intention toward AIAD? Our findings offer insights for design practices and tool developers on how to improve designers’ intention to use AIAD and depict implications for academic research and practical application in this field.

## 2 Literature review and research hypotheses

### 2.1 Artificial Intelligence-Aided Design (AIAD)

Recently, the application of artificial intelligence (AI) in design has been steadily increasing, and its role in aiding design is becoming more prominent. AI refers to computer technologies that can ‘make predictions, suggestions, and decisions impacting reality or virtual reality’ by giving machines a set of human-defined goals (Yeung, 2020). Moreover, AIAD emerges as an advanced interdisciplinary method that integrates AI technologies, especially deep-learning models and machine-learning algorithms, into the design procedures in diverse fields such as architecture (Li et al., 2023), graphic design (Liu, 2023), and engineering (Ao et al., 2023). By automating complex tasks, generating innovative solutions, and optimizing design elements, AIAD has enhanced creativity, efficiency, and accuracy (Pop and Schricker, 2023; Li et al., 2023; Liu, 2023; Guljajeva and Canet Sola, 2023). With the help of AIAD, designers can simulate and analyze a large amount of data, make real-time predictions, and optimize design outcomes. Furthermore, AIAD has transformed the conventional working procedures (Patel et al., 2024), opening avenues for unprecedented possibilities in design. For instance, Shin et al. (2024a,b) explained the design of AI systems by investigating how users perceive and understand fairness and transparency in the context of the Over-the-Top (OTT) platform. AIAD helps optimize design, generate inspiration, and develop intelligent models. Generative adversarial networks (GAN) are one of the most widely applied AI technologies in design (Gui et al., 2020), which are frequently integrated into supporting tools of design to generate inspiration, such as in designing the user interface (UI) (Mozaffari et al., 2022; Bunian et al., 2021), image stylization (Chen et al., 2018; Isola et al., 2017), craft arts (Deng and Chen, 2021), and virtual terrain (Guérin et al., 2017). For example, Mozaffari et al. (2022) proposed a type of GAN based on a particular style to generate a series of diverse and lumped UI examples. The CartoonGAN, presented by Chen et al. (2018), is a GAN specifically designed for cartoon stylization, capable of producing top-notch cartoon-style images from real-life photographs. Variational autoencoder (VAE) is another commonly used generative model. Huang (2018) introduced IntroVAE to synthesize high-resolution photos. Shi et al. (2020) introduced a tool designed to assist users in depicting emotional expressions for storyboarding, employing input strokes from the user. Some researchers integrated VAE with machine learning strategies of modern game design to generate new game maps (Mak et al., 2023). A convolutional neural network (CNN) is a deep learning architecture utilized in AI-powered design, drawing inspiration from the visual perception mechanisms observed in nature’s living creatures (Gu et al., 2018). In Gatys et al. (2016) proposed the first CNN-based model to support neural style transfer (NST) (Shi et al., 2023). This model is capable of rendering images by combining the image content with art drawing styles. For instance, Gatys et al. (2017) combined the style information of images from multiple sources via NST to build a new style with perceived attractiveness within the existing style. They also showcased how to apply the introduced control measures to the recent fast neural style transfer methods. Shi et al. (2023) found that NST is integrated into application tools for design both in the academic world (Champandard, 2016; Reimann et al., 2018; Siarohin et al., 2019) and the industry (Nikolaev et al., 2019). Besides visual outputs of images, AIAD is also employed to generate designs via text inputs. These applications include DALL-E, Imagen, Midjourney, and DreamStudio.

### 2.2 The Theory of Planned Behavior (TPB)

**Figure 1:**
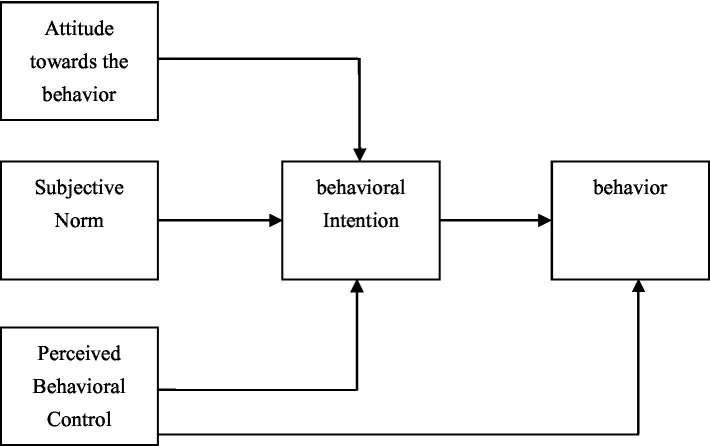
The Theory of Planned Behavior.

The Theory of Planned Behavior (TPB) is a primary theory on the antecedents of behavioral intention. It extended the earlier Theory of Reasoned Action (TRA) by including perceived behavioral control (PBC) as a variable, providing a more comprehensive understanding of individual control behaviors. According to TPB, an individual engages in a specific behavior based on their behavioral intention, which is influenced by attitudes, subjective norms, and perceived behavioral control (Figure 1). This research employed TPB to delve into the antecedents of designers’ intention to use AIAD, as TPB showcases great explanatory power in predicting the adoption of AI technological applications (Sohn and Kwon, 2020; Mohr and Kühl, 2021). TPB posits that attitudes toward behaviors are the positive or negative evaluations of specific behaviors. If one person makes a positive evaluation of a particular behavior, this person tends to have a greater intention to perform this behavior. Subjective norms pertain to the social pressures individuals perceive regarding the extent to which most people support or oppose a particular behavior. These pressures originate from significant individuals or groups who can influence a person’s decision to partake in the behavior (Ajzen, 1985). The higher the approval level, the higher the behavioral intention. Perceived behavioral control refers to an individual’s perception of the ease or difficulty involved in performing a specific behavior (Ajzen, 1985).

Numerous studies have investigated the factors influencing the intention to adopt technologies and have suggested that attitude is a crucial predictive factor. For instance, a survey of 409 university students discovered that their attitudes toward technology are significantly correlated with their behavioral intentions (Wang Q et al., 2022). Many prior investigations have adopted the various constructs of TPB to explain users’ intention to adopt technologies, and their hypotheses were validated in multiple fields such as e-commerce (Ozkan and Kanat, 2011), intelligent products (Jang and Noh, 2017; Sohn and Kwon, 2020; Song et al., 2018), marketing strategies (Choe et al., 2021). There are also studies focusing on AI assistance. Nevertheless, few researchers have touched upon the realm of AIAD employing TPB.

### 2.3 Technology Acceptance Model (TAM)

**Figure 2:**
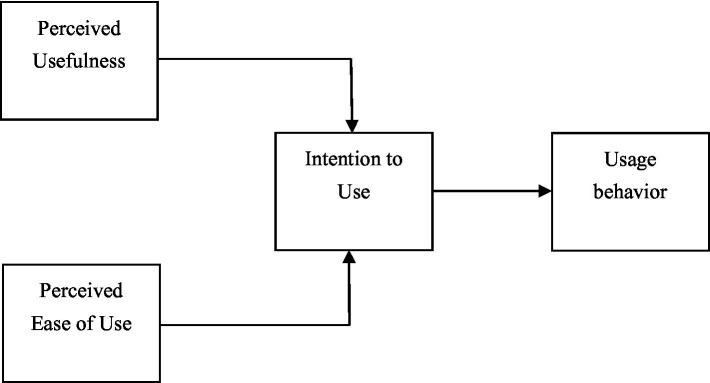
The Technology Acceptance Model.

The Technology Acceptance Model (TAM) emphasizes the impact of an individual’s perceptions of a technology’s usefulness and ease of use on their acceptance of that technology. User’ attitudes toward new technologies are substantiated to be vital to their intention to use these technologies (Davis, 1989). TAM explores the determinants of user acceptance of computer technologies, which can be extended to explain users’ behavioral intentions toward computer technologies. Some researchers believe that users’ perception and understanding of AI are determined by their cognition and the heuristic process (Shin et al., 2024a,b). TAM points out that ‘perceived usefulness’ and ‘perceived ease of use’ are two essential factors influencing user acceptance of technologies (Figure 2). Perceived usefulness refers to a user’s perception that utilizing a specific technology would enhance their efficiency. Perceived ease of use refers to a user’s perception that utilizing a particular technology would require minimal effort (Davis, 1989). Users’ cognitive processes are paramount in understanding AI’s operational mechanisms and decision-making processes and how these systems align with users’ expectations (Shin et al., 2024a,b). To this end, when users interact with AI systems, they can initiate a heuristic processing to evaluate the usefulness and ease of use of AI via clues such as transparency, fairness, and credibility (Shin et al., 2024a,b). In the context of burgeoning AI technologies, adopting AIAD tools, such as Midjourney and Stable Diffusion, has become increasingly prevalent among designers, university faculties, and students. In recent years, plenty of research on AIAD employed TAM to study the intention to adopt AI technologies in various contexts, including AI painting (Du et al., 2023), smart farming (Mohr and Kühl, 2021), intelligent products (Sohn and Kwon, 2020; Ponzoa et al., 2021; Song et al., 2018), architecture (Na et al., 2022; Lin and Xu, 2022), and e-commerce (Wang et al., 2023). Prior to interacting with AI systems, it is essential for users to integrate their cognitive process with a heuristic method to perceive them (Shin et al., 2024a,b). Thus, the adoption of AIAD has profound connections with users’ perceptual cognition of AIAD and their reliance on heuristic methods to thrive in the AI-driven design environment.

Both TPB and TAM stem from the Theory of Reasoned Action and they share two constructs: attitudes and behavioral intentions, although their focal points are slightly different (Liao et al., 2023). TPB deals with the impact of a person’s attitudes, subjective norms, and perceived behavioral control on behavioral intention, which is widely applied in exploring the general behaviors of individuals. The particularity of AI technologies makes it inadequate to employ a single theory of TPB to explain designers’ intention to use these technologies. Meanwhile, TAM emphasizes the impacts of perceived usefulness and perceived ease of use of new technologies on user acceptance. However, using TAM alone is also insufficient to fully explain the behavioral intention for AIAD as it neglects AIAD features, and TAM was initially established to improve the system design that can enhance the efficiency of employees within an organization. It does not involve other people’s influence on the users’ attitudes and behaviors (Rauniar et al., 2014). In the present study, AIAD involves general behavior (designing with the aid of AI) and particular technologies (AI-based tools). To this end, we believe that TPB and TAM are theoretically compatible and complementary in discussing designers’ behavioral intention to use AIAD.

### 2.4 Antecedents of behavioral intentions

#### 2.4.1 Attitudes toward behaviors

Attitude toward a behavior is defined as ‘the preference evaluation of a particular behavior and the judgment of the possible outcome of engaging in this behavior.’ It is believed to be the critical factor influencing people’s behavioral intentions (Ajzen, 1985; Ajzen, 1991). Many empirical studies have discovered that attitudes toward behaviors are positively correlated with their behavioral intentions (Aldammagh et al., 2021; Chen et al., 2019; Choe and Kim, 2018). Some scholars have suggested that one’s attitude toward a technology is a key prerequisite for adopting the technology (Nie et al., 2020; Faham and Asghari, 2019; Sohn and Kwon, 2020). For instance, Sohn and Kwon (2020) and Mohr and Kühl (2021) revealed that users’ attitudes toward innovative technologies are the key factor in predicting their intention to adopt them. Thus, based on this premise, this research proposes the following hypothesis:

H1: Users’ attitudes toward AIAD are positively correlated with their intention to use AIAD.

#### 2.4.2 Subjective norms

Subjective norms refer to the ‘social pressure regarding the decision to engage in a specific behavior,’ which is a key determinant of human behavior (Ajzen, 1985). The greater endorsement individuals gather from important people or groups surrounding them, the higher their intention to perform the behavior of interest. Prior research has indicated that subjective norms positively impact behavioral intention in different contexts, such as e-learning (Chu and Chen, 2016), patent application (Lin and Yu, 2018), and drone food delivery (Choe et al., 2021). In the context of AI, Sohn and Kwon (2020) discovered that subjective norms are critical in impacting the purchase behavior of AI-based intelligent products. They also pointed out that AI is a kind of technology that people are interested in but lack practical experience with. In this case, when deciding whether to adopt certain AI-based intelligent products, they would be subject to the influence of other people’s opinions. In this regard, the following hypothesis is posited:

H2: Subjective norms are correlated in a positive manner with the intention to use AIAD.

#### 2.4.3 Perceived behavioral control

In TPB, perceived behavioral control is an individual’s judgment of how challenging or straightforward it is to execute the behavior of interest (Ajzen, 1985; Ajzen, 1991), encompassing both internal and external factors like abilities and resources. Individuals with higher perceived control over resources and abilities toward the behavioral goal are more likely to perform the behavior. Evidence from certain studies suggests that perceived behavioral control significantly influences the intention to perform the behavior in an apparent and positive manner (Nie et al., 2020; Choe et al., 2021; Mohr and Kühl, 2021). Specifically, these studies suggest that perceived behavioral control serves as a positive predictor of the adoption of e-learning, drone food delivery, and smart farming systems. In the context of AIAD, an individual’s perceived behavioral control can positively predict their intention to use AIAD. To this end, we put forward the following hypothesis:

H3: Perceived behavioral control is positively correlated with the intention to use AIAD.

### 2.5 Perceived usefulness, perceived ease of use, and attitudes

Perceived usefulness refers to how much one perceives new technology can enhance efficiency (Venkatesh and Davis, 2000), which is also explained as the subjective probability of potential users (Chatterjee et al., 2021). Perceived ease of use is how much an individual believes that using a new technology can be effortless without dedicating considerable time to learning (Sun et al., 2022). Perceived ease of use and perceived usefulness precede the intention to use these technologies (Venkatesh and Davis, 2000; Venkatesh et al., 2003). Other research has suggested that perceived usefulness and ease of use have positive and notable impacts on the intention to use these technologies (Liu et al., 2010; Choe et al., 2021; Cao et al., 2023). In the context of AI, research reports have pointed out that perceived usefulness and ease of use are positively correlated with user acceptance of the AI-based painting tool (Shen et al., 2023; Du et al., 2023). We thus hypothesize that:

H4: Perceived usefulness is positively correlated with attitudes toward AIAD.

H5: Perceived ease of use is positively correlated with attitudes toward AIAD.

### 2.6 Knowledge level

Most extant literature believes that attitudes exert a positive influence on intention (Gurlitt and Renkl, 2010). Knowledge level usually refers to the depth and breadth of the knowledge one masters in a specific field or multiple fields, which includes the understanding and application capacity of facts, notions, principles, skills, and experience. According to Alba and Hutchinson (1987), knowledge comprises familiarity and expertise. Familiarity refers to the capability of understanding objects’ statuses via knowledge, and expertise is defined as the capability of performing tasks by applying knowledge. Previous research has demonstrated that users’ knowledge level significantly influences their consumption behaviors (Gurlitt and Renkl, 2010). Venkataraman et al. (1990) posited that consumer knowledge is the unique information one person has over a specific theme, which enables individuals to identify opportunities. In the domain of AIAD, consumer knowledge involves the degree of familiarity with AIAD and professional knowledge in AIAD-related fields (Schreier and Prügl, 2008). According to Swaminathan (2003) and Barrutia and Gilsanz (2013), consumer behavior depends on their knowledge of the goods of interest. Therefore, we predict that designers’ knowledge level can directly or indirectly influence their intention to use AIAD. Wang Y. et al. (2022) discovered that consumers with a higher level of knowledge of intelligent service robots tended to reduce their usage of them. Zhang et al. (2022) uncovered that users with a higher level of knowledge showed a reduced intention to pay for AIAD. To this end, we hypothesize that:

H6: Knowledge level negatively moderates the influence of attitudes toward AIAD on the intention to use AIAD.

### 2.7 Our research

**Figure 3:**
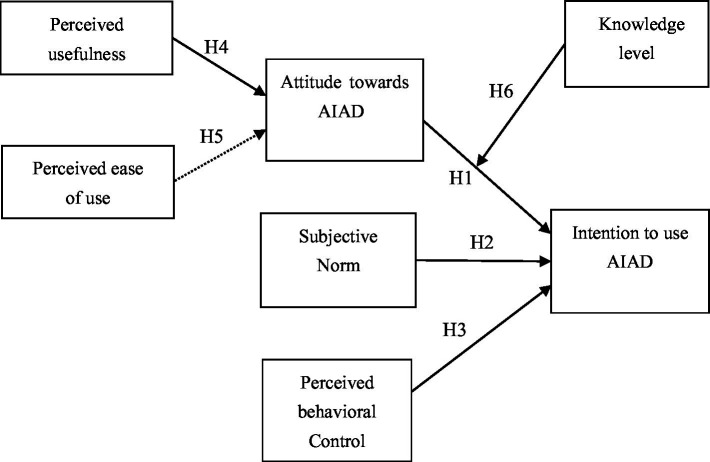
The research model.

Employing the integrated theoretical framework of TPB and TAM, this research explores the influences of designers’ attitudes toward AIAD, subjective norms, and perceived behavioral control on their intention to use AIAD and the influences of perceived usefulness and ease of use on attitudes. Moreover, the moderating impact of knowledge on attitudes’ role in the intention to use is investigated. Figure 3 presents the research model, in which attitudes, subjective norms, perceived behavioral control, knowledge level, perceived usefulness, and perceived ease of use are latent variables, and the intention to use AIAD is the observable variable.

## 3 Methods

### 3.1 Participants

We conducted an empirical study to test the hypotheses via an online survey. Convenience sampling was utilized to gather the data. It is a non-probability sampling method that researchers would adopt based on the principle of convenience in sampling. The target group of respondents is usually individuals or groups that are easy to reach (Stratton, 2021). The research participants are graduate and undergraduate students majoring in design from various regions across China, as well as design practitioners. An online survey was created and distributed to participants for voluntary and anonymous completion. The process was conducted online. The first section of the survey introduces the current research and the research objective to ensure that participants are familiar with or have used AIAD. We received 392 valid responses, of which 45.40% were undergraduates majoring in art and design, 20.66% were graduates majoring in art and design, 27.55% were practitioners in design, and 6.37% were classified as others. The statistics of participants and their familiarity with AIAD are displayed in Table 1.

**Table 1:** Demographic information of the participants.

	Items	Number	Percentage
Occupation	Undergraduate students	178	45.40%
	Graduate students	81	20.66%
	Workers	108	27.55%
	Others	25	6.37%

### 3.2 Instrument development

This research employs a structured questionnaire comprising two sections. The initial section includes inquiries to gather participant demographics and their acquaintance with AIAD.

In the second part, we designed 21 items to gather empirical evidence to test the six latent variables of the research model. Each item was either sourced or adapted from previous literature, ensuring satisfying reliability and validity. A five-point Likert scale was utilized, ranging from ‘strongly disagree (1)’ to ‘strongly agree (5)’.

All variables from this research were adapted from previously verified scales to fit in with the context of AIAD. In particular, the subscales for the four constructs of TPB (attitudes, subjective norms, perceived behavioral control, and behavioral intention) were sourced from the results of prior research (Chu and Chen, 2016; Wang Q. et al., 2022), with each construct being measured through three items. The measures for perceived usefulness and perceived ease of use in TAM were drawn from the research outcomes of Davis (1989) and Choe et al., 2021, each comprising three items for each construct. The scale for knowledge level was modified based on the one in Trzebinski et al.’s (2023) investigation, with three items for the construct. Subsequently, 20 professionals with different design backgrounds were invited to participate in the pretest. The final questionnaire was determined after a modification based on their opinions.

### 3.3 Data analysis

This study adopted AMOS, SPSS, and PROCESS for data analysis. The analysis process includes three steps. First, Confirmatory Factor Analysis (CFA) was performed to test the reliability and validity of the scale. Next, Structural Equation Modeling (SEM) was adopted to verify the research hypotheses. Lastly, PROCESS was utilized to test the moderating effect of knowledge level.

## 4 Findings

### 4.1 Measurement model

The reliability of the research was assessed using the value of Cronbach’s alpha for factor loading and constructs. The CFA results (Table 2) suggested that the values of composite reliability (CR) and Cronbach’s alpha of all factors exceeded the thresholds, indicating fair test quality. Detailed outcomes are presented in Table 3. The CR for every construct was observed to range from 0.802 to 0.894, surpassing the widely accepted minimum threshold of 0.60 (Hair Jr. et al., 2014); the value of Cronbach’s alpha of each construct was found to fall within the range of 0.790–0.894, surpassing the threshold of 0.70; and the average of variance extracted (AVE) of each construct ranged from 0.576 to 0.738, surpassing the threshold of 0.50 as stipulated by Fornell and Larcker (1981). Furthermore, the square root of the AVE for each construct surpassed the correlations between this construct and other constructs. All the above results indicated that the discriminant validity and convergent validity were acceptable according to the standards set by Fornell and Larcker (1981). Moreover, the research model’s goodness of fit was calculated (*X*^2^ = 495.895, df = 247, *X*^2^/df = 2.952, TLI = 0.928). According to Hair Jr. et al. (2014), a model is considered a good fit if the root-mean-square error of approximation (RMSEA) and the standardized residual mean root (SRMR) are under 0.05, and the Comparative Fit Index (CFI) and Tucker-Lewis index (TLI) are beyond 0.95. For an acceptable fit, RMSEA and SRMR should be under 0.08, and CFI and TLI should surpass 0.90. In the present research, the model demonstrates a satisfactory fit (CFI = 0.942, SRMR = 0.048, RMSEA = 0.071).

**Table 2:** Results of construct validity and reliability analysis.

Latent variable	Measurement variable	Mean	Std. Dev.	Factor loadings	*α*
ATT	ATT1	4.128	0.760	0.851	0.894
	ATT2			0.866	
	ATT3			0.861	
SN	SN1	3.828	0.737	0.489	0.790
	SN2			0.918	
	SN3			0.872	
PBC	PCB1	3.625	0.830	0.755	0.836
	PCB2			0.827	
	PCB3			0.802	
PU	PU1	4.043	0.753	0.868	0.888
	PU2			0.802	
	PU3			0.884	
PEOU	PEOU1	3.811	0.727	0.742	0.831
	PEOU2			0.802	
	PEOU3			0.818	
KL	KL1	3.513	0.867	0.817	0.862
	KL2			0.860	
	KL3			0.802	
IN	IN1	3.948	0.692	0.840	0.803
	IN2			0.727	
	IN3			0.703	

ATT, attitude; PBC, perceived behavioral control; SN, subjective norms; PU, perceived usefulness; PEOU, perceived ease of use; KL, knowledge level; IN, intention.

**Table 3:** The results of discriminant validity and convergent validity.

Constructs	CR	AVE	ATT	SN	PB	PU	PEOU	KL	IN
ATT	0.894	0.738	0.859						
SN	0.818	0.614	0.557	0.784					
PBC	0.837	0.632	0.470	0.445	0.795				
PU	0.888	0.726	0.764	0.618	0.554	0.852			
PEOU	0.830	0.621	0.534	0.625	0.587	0.670	0.788		
KL	0.866	0.683	0.379	0.493	0.728	0.457	0.490	0.826	
IN	0.802	0.576	0.562	0.547	0.597	0.564	0.616	0.542	0.759

ATT, attitude; PBC, perceived behavioral control; SN, subjective norms; PU, perceived usefulness; PEOU, perceived ease of use; KL, knowledge level; IN, intention.

### 4.2 Structural model

The goodness of fit of the model via SEM is as follows: *X*^2^ = 572.220, df = 125, *X*^2^/df = 4.578, CFI = 0.904, TLI = 0.883, RMSEA = 0.096. According to relaxed standards, the model’s goodness of fit is acceptable (Hair Jr. et al. 2014; Fornell and Larcker, 1981).

**Figure 4:**
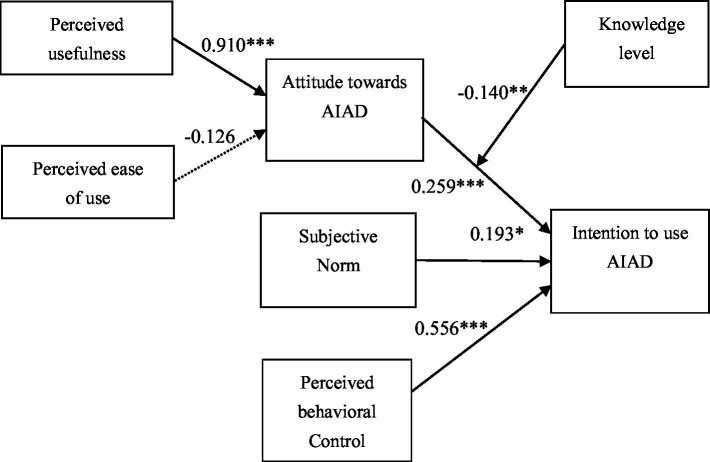
The research model with its path coefficients. ***p < 0.001, **p < 0.01, *p < 0.05.

Table 4 displays the test results. All hypotheses but H5 were supported by the data. Attitudes (*b* = 0.259, *p* < 0.001), subjective norms (*b* = 0.193, *p* < 0.01), and perceived behavioral control (*b* = 0.556, *p* < 0.001) are significantly and positively correlated with the intention to use AIAD. While perceived usefulness (*b* = −0.126, *p* > 0.05) has a significant and positive correlation with attitudes toward AIAD, perceived ease of use (*b* = −0.126, *p* > 0.05) has no significantly positive correlation with attitudes. Moreover, knowledge level (*b* = −0.140, *p* < 0.01) negatively moderates the impact of attitudes on the intention to use AIAD. The research model, along with its path coefficients, is illustrated in Figure 4.

**Table 4:** The results of hypotheses testing.

Hypotheses	Hypothesized	*b*	S.E	*t*	Result
H1	ATT→IN	0.259	0.058	4.240***	Supported
H2	SN→IN	0.193	0.047	2.929**	Supported
H3	PBC → IN	0.556	0.064	8.101***	Supported
H4	PU→ATT	0.910	0.072	12.758***	Supported
H5	PEOU→ATT	−0.126	0.073	−1.710	Rejected
H6	KL’s Moderation	−0.140	0.037	−3.791**	Supported

ATT, attitude; PBC, perceived behavioral control; SN, subjective norms; PI, perceived interest; KL, knowledge level; IN, intention.

***p < 0.001, **p < 0.01, *p < 0.05.

## 5 Discussion

Employing the theoretical framework integrating TPB and TAM and considering the influence of knowledge level, this study explores the determinants of designers’ intention to use AIAD, specifically the direct influences of attitudes, subjective norms, and perceived behavioral control on designers’ intention to use AIAD and the influences of perceived usefulness and perceived ease of use on attitudes. Moreover, we explore the moderating impact of knowledge level on the attitude-to-intention process.

### 5.1 TPB explains the intention to use AIAD

First, the study findings show that attitudes, subjective norms, and perceived behavioral control are strongly and positively correlated with the intention to use AIAD, validating the hypotheses in TPB (Ajzen, 1991). Specifically speaking, perceived behavioral control shows the most substantial influence, followed by attitudes and subjective norms. The significantly positive correlation between attitudes and the intention to use aligns with the research findings of previous research (Chai et al., 2020). Subjective norms are markedly and positively correlated with the intention to use, aligning with prior research (Fan and Jiang, 2024) but misaligning the research findings of Chai et al. (2020), which showed that subjective norms did not directly predict the intention. Our findings suggest that designers are subjected to the influence of other people’s opinions in the adoption of AIAD. This inconsistency could be due to the different measurement models and participants’ ages. The research subjects in Chai et al. (2020) study are mainly secondary school students under 18, while ours are adults over 20. Moreover, in the domain of AI, compared with secondary school students and college students, designers are exposed to AI in a more professional environment, and they have more opportunities to exchange ideas and interact with classmates, colleagues, teachers, or experts on AI. Given this background, our research participants are more likely to be subjected to the influence of other people’s opinions, and they may tend to follow suit with the mainstream trend. Furthermore, our research participants have more professional knowledge and cumulative experience in design and a deeper understanding of design tools and technologies. To this end, they are more easily influenced by the opinions of peers or experts. Perceived behavior control has a positive correlation with the intention to use, aligning with the observations made by Zhou et al. (2024). Designers’ evaluation of their mastery of knowledge and skills impacts their intention to use AIAD.

### 5.2 Influences of perceived usefulness and ease of use on attitudes

Perceived usefulness positively impacts the effect of attitudes on the intention to use. Evidence of this analysis has been provided in numerous prior studies (Chai et al., 2020; Shen et al., 2023; Cao et al., 2023; Gao et al., 2024). This finding indicates that designers would perceive AI tools as of greater usefulness when these tools can improve the efficiency and creativity of design tasks. Thus, they would adopt more positive attitudes toward AIAD.

Contrary to our expectations, our analysis reveals that perceived ease of use negatively impacts the influence of attitudes on the intention to use AIAD. This result diverges from prior research (Cao et al., 2023; Choe et al., 2021; Gao et al., 2024) but aligns with the research findings of Nathania and Anandya (2021). In the context of AIAD, individuals using AIAD are primarily design professionals equipped with advanced skills and capabilities. They tend to exhibit a higher degree of autonomy when deciding whether to use AIAD. For them, the complexity of tools is not necessarily a deterrent but could be a stimulant to flexibility and improved functionality (Fan and Jiang, 2024). Despite the anticipation of the difficulty of use the AI tools, designers care more about the outcome of using these tools instead of the ease of use. Besides, as AI is going to be the major development direction of many industries, especially in design, using AI-based technologies will significantly improve the efficiency of designers and students majoring in design (Cao et al., 2023). Therefore, even without perceived ease of use, designers’ attitudes toward AIAD can still be positive and they may still adopt AIAD.

### 5.3 The moderating effect of knowledge level

Our research findings suggest that knowledge has a negative moderating impact on the influence of attitudes on the intention to use, aligning with previous research (Zhang et al., 2022; Fu and Elliott, 2013). This finding indicates that the higher the designers’ knowledge level of AIAD, the weaker their intention to use AIAD. Designers with AIAD literacy could cast doubt on the use of AIAD as they have a deeper understanding of the limitations of these tools. For instance, some AIAD tools depend on massive data input for training and learning, which could give rise to reduced performance in the case of data scarcity and inadequacy and further lead to unsatisfying design outcomes, thus lowering their intention to use AIAD.

### 5.4 Practical implications

Our research results bring practical significance to the adoption of AIAD. In light of the positive correlation between subjective norms and the intention to use AIAD, developers of AI-based design tools can invite experts or pundits to evaluate their products, give professional certification, or make recommendations to increase designers’ trust in these tools and the intention to use them. Besides, developers can also publicize the advantages of AIAD in professional communities and communication platforms, such as boosting design efficiency and creativity and optimizing design procedures. Spontaneous recommendations can positively impact designers’ subjective norms and enhance their exposure to a positive atmosphere of using AIAD, thereby enhancing their intention to use AIAD.

Perceived behavioral control positively impacts the intention to use AIAD, demonstrating that designers’ belief and evaluation that they can control or influence AIAD tools will make them more inclined to use AIAD tools. Given this analysis, algorithmic detection companies of AIAD tools can reduce the barriers to learning and using AIAD tools by optimizing the interface design and providing clear operation procedures and video tutorials, thereby facilitating designers’ mastery of these tools and evoking a sense of control within them.

The present study reveals that perceived usefulness has a positive influence on the behavioral intention to use AIAD, signifying that designers’ intention to use AIAD can be boosted if designers perceive more interest in using AIAD. In future practices, algorithmic detection companies of AIAD tools can introduce the functions and features of AIAD tools clearly and showcase some successful cases and practical applications to strengthen users’ trust in the usefulness of AIAD. Furthermore, there should be more beginner guides to help new users to get started quickly. For example, they can enrich the interaction part of user guides in a way similar to the electronic game guides and offer a more accurate instruction feedback experience. They can also apply the user-centered approach to the customization services (Shin et al., 2024a,b). Moreover, AIAD tools can provide flexible customization services that enable designers to customize the use of these tools based on their preferences to enhance the perceived usefulness of AIAD.

Moreover, our research results demonstrate that knowledge level plays a negative moderating role. Designers with a higher knowledge level of AIAD know more about the limitations of these tools. Algorithmic detection companies of AIAD tools can improve the transparency and explainability of tools. By offering the perceptible decision-making process of algorithmic tools to designers, designers can better understand how AIAD tools work and where their limitations come from, thereby improving their sense of trust and acceptance. To this end, algorithmic detection companies can collect advice from designers regularly to improve the functions and performance of these tools. By optimizing the flaws of AIAD tools, their practicability and user experience can be enhanced, thus improving designers’ intention to use them.

### 5.5 Theoretical implications

First, this study established a theoretical framework by integrating TPB and TAM, which captures the multi-dimensional factors influencing designers’ acceptance of AIAD. By integrating these variables, we can further understand how the conventional determinant factor (attitudes) interacts with the specific technological factor (perceived usefulness). Moreover, the involvement of perceived usefulness impacts designers’ intention to use AIAD and influences their cognitive process and attitudes in decision-making. These influences indicate that perceived usefulness is a precondition of accepting new technologies and determines how designers interact with technologies while using them. To this end, our research verifies the significant correlations among variables via the comprehensive framework integrating TAM and TPB and enriches the extant literature. Furthermore, it proffers insights into addressing the complexity perceived by designers and new perspectives for adopting AIAD in relevant industries.

Second, this study substantiates the moderating effect of knowledge level in AIAD. It was found that designers with higher knowledge of AIAD displayed lower intention to use AIAD as these designers could know more about their limitations and hold a suspicious attitude toward the authenticity and accuracy of AIAD-generated content, thus mitigating their confidence in using these tools. For example, some AIAD tools rely on a large amount of data for training and learning. These data and algorithms are subject to non-transparency and data pollution, and their accuracy has not been verified (Shin and Valente, 2020; Shin et al., 2024a,b), which could lead to the performance decline of tools in cases of data scarcity and incompleteness, further trigger unsatisfactory design results and lower designers’ intention to use AIAD tools.

## 6 Conclusion

From the perspectives of TPB and TAM, this research seeks to understand the underlying mechanism governing designers’ adoption of AIAD. The empirical evidence garnered from our investigation demonstrates that designers’ attitudes toward AIAD, subjective norms, and perceived behavioral control have significant and positive influences on their intention to use AIAD. Moreover, it is substantiated that the perceived usefulness of AIAD significantly and positively impacts designers’ attitudes toward AIAD and thus indirectly influences their intention to use AIAD. Knowledge level negatively moderates the influence of attitudes on the intention to use AIAD. The present study offers three contributions. First, we extend extant intention-related literature by emphasizing the critical roles of TPB, TAM, and knowledge level. The introduction of knowledge level as a moderating variable provides reasonable explanations for the contradictory results of previous research exploring the correlation between AIAD and perceived ease of use. Second, to our knowledge, the study represents the first attempt to explore the impact of knowledge level on designers’ intention to use AIAD. Our findings substantiate that perceived ease of use can exert no impact on the adoption of AIAD. Furthermore, we expand the literature on studying the influence of perceived usefulness on attitudes toward a behavior by verifying the absolute importance of perceived usefulness in impacting designers’ attitudes toward AIAD.

Despite the valuable contributions mentioned above, the limitations of this study still need to be addressed. First, our research subjects are mainly people with design backgrounds, and we did not divide them into groups according to their age and gender, making our research findings a little bit general as it is plausible that designers at different ages may exhibit different characteristics. Next, the size of the sample in this study is relatively small. Moving forward, we will explore the influences of age and gender and expand our sample size by collecting data from multiple channels to enrich our understanding of designers’ adoption of AIAD.

## Data Availability

The datasets presented in this article are not readily available because data may be obtained by contacting the corresponding author. Requests to access the datasets should be directed to Xiangnan Cao, 742674274@qq.com.
